# Hypoxia Promotes Migration and Induces CXCR4 Expression via HIF-1α Activation in Human Osteosarcoma

**DOI:** 10.1371/journal.pone.0090518

**Published:** 2014-03-11

**Authors:** Mingjun Guo, Chengkui Cai, Guangyi Zhao, Xiuchun Qiu, Haien Zhao, Qiong Ma, Liying Tian, Xuelian Li, Yunsheng Hu, Bo Liao, Baoan Ma, Qingyu Fan

**Affiliations:** 1 Department of Orthopaedics, Tangdu Hospital, Fourth Military Medical University, Xi'an, Shaanxi, People's Republic of China; 2 Department of Orthopaedics, Bethune International Peace Hospital, Shijiazhuang, Hebei, People's Republic of China; 3 Department of Anesthesiology, Tangdu Hospital, Fourth Military Medical University, Xi'an, Shaanxi, People's Republic of China; 4 Department of Surgery, Xi'an Hospital of TCM, Shaanxi, People's Republic of China; H.Lee Moffitt Cancer Center & Research Institute, United States of America

## Abstract

**Background:**

Cellular adaptation to a hypoxic microenvironment is essential for tumor progression and is largely mediated by HIF-1α through coordinated regulation of hypoxia-responsive genes. The chemokine SDF-1α and its unique receptor CXCR4 have been implicated in organ-specific metastases of many cancers. In this study, we investigated the response of osteosarcoma cells to hypoxia and the expression of CXCR4 and HIF-1α in human osteosarcoma specimens and explored the roles of CXCR4 and HIF-1α in the cell migration process.

**Methodology/Principal Findings:**

We performed immunohistochemistry, immunocytochemistry, quantitative real-time PCR, Western blots and fluorescent reporter assays to evaluate the correlation between CXCR4 and HIF-1α expression in human osteosarcoma specimens or SOSP-9607 cells under normoxic and hypoxic conditions. Transwell assays were used to assess cell migration under different conditions. Exposure of SOSP-9607 cells to hypoxic conditions resulted in significantly increased migration. When SOSP-9607 cells were subjected to hypoxic conditions, the mRNA and protein levels of CXCR4 were significantly increased in a time-dependent manner. Moreover, siHIF-1α significantly decreased the mRNA and protein levels of CXCR4 under hypoxia, whereas pcDNA-HIF-1α significantly increased the mRNA and protein levels of CXCR4 under normoxia. A luciferase reporter gene study showed that siHIF-1α reduced pGL3-CXCR4 luciferase activity. Furthermore, coexpression of HIF-1α and CXCR4 was significantly higher in patients with distant metastasis compared with those without metastasis.

**Conclusions/Significance:**

The hypoxia-HIF-1α-CXCR4 pathway plays a crucial role during the migration of human osteosarcoma cells, and targeting this pathway might represent a novel therapeutic strategy for patients suffering from osteosarcoma.

## Introduction

Osteosarcoma (OS) is a highly malignant, aggressive tumor of the bone, with a peak incidence in the second and third decades of life. It accounts for approximately 45% of all bone sarcomas [1]. Although up to 50–70% 5-year survival rates can be achieved by multimodal therapy, a large group of patients are still left with a poor prognosis due to a lack of effective treatment options [2]. With the rapid expansion of knowledge concerning stem cell biology, emerging evidence suggests that OS should be regarded as a type of differentiation disease caused by genetic and epigenetic changes that interrupt the differentiation of mesenchymal stem cells into osteoblasts. OS is a locally destructive tumor with a high potential for metastasis [3]. Prognostic evaluation of patients with OS is limited to clinical parameters, whereas molecular markers of tumor aggressiveness have yet to be definitively identified [4]. OSs with metastases present worse clinical outcomes than those without metastases. Thus, more effective treatments and/or personalized therapeutic methods (i.e., treatments according to specific gene or protein profiles) are needed for patients with OS with pulmonary metastases [5].

CXCR4 plays a key role in the metastatic homing of tumor cells to organs expressing a high level of its ligand, SDF-1 [6]. To date, CXCR4 is one of the most common chemokine receptors demonstrated to be overexpressed in human cancers, with overexpression reported in more than 23 cancer types, including breast cancer, ovarian cancer, melanoma and prostate cancer [7]. HIF-1 is the key regulator of cellular response to hypoxia and plays a central role in the control of tumor growth [8].

Molecules induced by hypoxia that promote survival, invasion and angiogenesis in the primary tumor may function similarly at the secondary site. In addition, hypoxia may upregulate proteins that mediate interactions with unique stromal cells in the secondary organ. It has been shown that tumor cells utilize distinct sets of genes when colonizing different organs. However, the method by which hypoxia influences organ-specific metastasis is unknown [9]. HIF-1 is a heterodimeric transcription factor that responds to oxygen concentrations in tissues and has been shown to upregulate CXCR4 expression. Thus, in hypoxic regions of expanding tumors, chemokine receptor levels might be increased to facilitate survival and escape from the primary tumor mass. In addition to facilitating distant metastasis, HIF-1 has been shown to induce CXCR4 in gliomas, leading to enhanced proliferation, resistance to apoptosis and local invasion [10]. Lu X recently found that hypoxia affects the gene signatures of both lung and bone metastasis in different ways in breast cancer: although hypoxia enhanced the expression of a large percentage of genes involved in lung metastasis, it also activated a more limited number of bone metastasis genes, such as CXCR4 and DUSP1 [11].

Previous studies have confirmed a relationship between CXCR4 and HIF-1α expression in different cancers [12]–[14]. The CXCR4 promoter includes four potential hypoxia-response elements (HREs) located within 2.6 kb upstream of the transcriptional start site and one at position −1.3 kb within the intron [15]. This result indicates that CXCR4 is a hypoxia-responsive gene. Therefore, hypoxia may affect the human OS cell migration process by altering the expression of CXCR4 via HIF-1α activation.

To test this hypothesis, we employed SOSP-9607 cells [16] to evaluate the effect of hypoxia on human OS. As an initial step, we observed that hypoxia enhanced the migration of SOSP-9607 cells and increased the mRNA and protein levels of CXCR4. In addition, we also demonstrated that the expression of CXCR4 induced by hypoxia may have occurred through the activation of HIF-1α. Finally, we evaluated the co-expression of CXCR4 and HIF-1α in human OS specimens. We believe this knowledge may lead to potentially important therapeutics for human OS.

## Materials and Methods

### Ethics statement

All research involving human tissue samples was approved by the Ethics Review Committee of the Fourth Military Medical University, Xi'an, Shaanxi, China (approval ID:2012042), and written informed consent was obtained from all participating patients.

### Cells lines and culture

Human OS cell line SOSP-9607 was established and maintained in our laboratory as previously described [16]. Cells were grown in RPMI 1640 medium (HyClone, USA) supplemented with 10% fetal bovine serum (FBS), 2 mM glutamine, 100 U/ml penicillin, and 100 mg/ml streptomycin at 37°C in 5% CO_2_ and 95% atmosphere. For hypoxic conditions, the cells were exposed to 2% O_2_ with 5% CO_2_ at 37°C. After incubation for the desired periods, the cells were harvested for subsequent experiments.

### Migration assays

The metastatic potential of cells was measured in 6.5 mm Transwells with 8.0 µm pore polycarbonate membrane inserts (Corning, USA) according to the manufacturer's instructions. SOSP-9607 cells were treated with hypoxia for 48 h and then starved in serum-free media overnight. SOSP-9607 cells transfected with siHIF-1α/NC-siRNA or siCXCR4/NC-siCXCR4 were similarly treated with hypoxia for 48 h and then starved in serum-free media overnight. The cells were then harvested and resuspended in migration medium (RPMI 1640 medium). A suspension of 3,000 cells in 100 µl migration medium was added to each top chamber. The cells maintained under normoxic conditions were used as a control. After the cells were incubated for 12 h, the non-migrating cells that remained on the upper surface were removed with a cotton swab. The cells that had migrated to the lower surface of the membrane insert were fixed with 4% paraformaldehyde for 30 min, permeabilized with 0.2% Triton X-100 at room temperature for 15 min, and then stained with 0.1% crystal violet for 5 min. The number of cells that migrated was counted under a light microscope from five random fields at a magnification of 100×. Three independent replicates were performed, and the results are given as the means ± standard deviation (SD).

### Quantitative real-time PCR (qRT-PCR)

Total RNA was extracted from SOSP-9607 cells, and cDNA was synthesized using AMV reverse transcriptase at 42°C for 10 min followed by 95°C for 2 min. ABI SybrGreen PCR Master Mix and ABI Step One Plus Real-Time PCR System (CA, USA) were used. The reagents were subjected to 95°C for 2 min and then subjected to 40 cycles of 95°C for 10 s and 60°C for 40 s. The primers for HIF-1α were: 5′-GACAGTACAGGATGCTTGCC-3′ (forward) and 5′-GCTGAATAATACCACTCACAACG-3′ (reverse). The primers for CXCR4 were: 5′-AATAAAATCTTCCTGCCCACC-3′ (forward) and 5′-CTGTACTTGTCCGTCATGCTTC-3′ (reverse). The primers for GAPDH were: 5′-TGGGTGTGAACCATGAGAAGT-3′ (forward) and 5′-TGAGTCCTTCCACGATACCAA-3′ (reverse). The relative quantity of real-time PCR products was analyzed using an ABI 7900HT software system.

### Western blot analysis

Equal amounts (40 µg) of protein lysate were separated by SDS-PAGE (Bio-Rad, USA) and transferred to a polyvinylidene difluoride (PVDF) membrane (Millipore, USA). The membranes were blocked with TBST containing 5% non-fat dry milk for 1 h and incubated with a mouse monoclonal primary antibody against HIF-1α (1∶300; ab113642, Abcam) or mouse monoclonal anti-CXCR4 (1∶300; ab58176, Abcam) and mouse monoclonal anti-β-actin (1∶1000; Sigma-Aldrich) overnight at 4°C. After washing three times with TBST, the membranes were incubated with horseradish peroxidase-conjugated anti-mouse secondary antibody (Santa Cruz, USA) for 2 h. The membranes were again washed three times in TBST, and the proteins were visualized using the enhanced chemiluminescence (ECL) Plus kit (GE Healthcare Bio-Sciences). β-actin was used as a control. The optical density of protein fragments was quantified using Quantity One software (Bio-Rad, USA).

### Immunocytochemistry

Cells were stained with antibodies to HIF-1α (1∶500; ab113642, Abcam), CXCR4 (1∶500; ab1671, Abcam) and multi-use secondary antibody (1∶1000; Dako, Ely, UK). The primary antibody was omitted in the negative control. Staining was visualized with the EnVision™ Peroxidase/DAB Rabbit/Mouse detection kit (Dako). All experimental procedures were performed according to the ICC (IF) protocol from Abcam.

### Synthetic small interfering RNAs (siRNAs), plasmid and transfection

Synthetic siRNAs specific for HIF-1α (siHIF-1α) and negative control siRNAs were designed and synthesized by GenePharma (Shanghai, China). The sequence for siHIF-1α was: 5′-GCCGCUCAAUUUAUGAAUATT/UAUUCAUAAAUUGAGCGGCTT-3′. The negative control siRNA (NC-siRNA) was of the sequence: 5′-UUCUCCGAACGUGUCACGUTT/ACGUGACACGUUCGGAGAATT-3′. Synthesized siHIF-1α was resuspended in RNase-free DEPC water to a final concentration of 20 µM. Cells were seeded at a density of 8×10^5^ per well in 60 mm culture dishes in complete medium and cultured overnight. Two hundred picomoles of siHIF-1α was mixed with Lipofectamine 2000™ reagent (Invitrogen, USA) according to the manufacturer's instructions. The transfected cells were then exposed to normoxic or hypoxic conditions. Forty eight hours after transfection, protein or total RNA was extracted from cells for subsequent experiments. The pcDNA plasmid carrying HIF-1α (pcDNA-HIF-1α) and random sequence (pcDNA-NC) was also purchased from GenePharma (Shanghai, China) and transfected into SOSP-9607 cells according to the manufacturer's instructions as described above.

### Luciferase reporter gene expression

The CXCR4 core promoter (−1594–+68 bp) was amplified by PCR using primers with sequences selected from the CXCR4 core promoter sequence (Forward: 5′-CGCGCTCGAGGTGACTCACGGTAAAGG-3′, Reverse: 5′-CGCGAAGCTTGGGTTGATTTCAGCACCT-3′). The cycling parameters were 95°C for 10 s as a pre-denaturing step, followed by 40 cycles of 95°C for 5 s, 55°C for 30 s, and 72°C for 10 min. PCR products were gel purified using a QIAEXII Gel Extraction Kit (Qiagen, Valencia, CA) and inserted into the PGL3-basic vector (Promega, Madison, WI) using HindIII and XhoI (New England Biolabs) as restriction sites; this vector was termed PGL3-CXCR4. The 1.663 kb CXCR4 promoter fragments of the pGL3 basic vector were confirmed by nucleotide sequence analysis. SOSP-9607 cells were plated into 6-well plates one day prior to transfection. Following confirmation of 70%–80% confluence, the cells were transfected with pGL3-Basic without a promoter (negative control) or pGL3-CXCR4 (normal plasmid). For cell transfections, SOSP-9607 cells were transiently transfected with 2 µg plasmid and 0.2 µg internal control plasmid pRL-TK using Lipofectamine 2000™ reagent according to the manufacturer's instructions. Plasmid pRL-TK was used as an internal control.

### Immunohistochemistry

Formalin-fixed and paraffin-embedded sections were stained for HIF-1α (1∶200; ab113642, Abcam) and CXCR4 (1∶200; ab58176, Abcam) using multi-use secondary antibody (1∶1000; Dako, Ely, UK). Staining was visualized with the EnVision™ Peroxidase/DAB Rabbit/Mouse detection kit (Dako). All experimental procedures were performed according to the IHC-P staining protocol of Abcam. Image acquisition was performed using an Olympus BX40 microscope with a 10× or 20× objective. Staining intensity was evaluated by systematically screening all slices and evaluating them according to an established 0–3 scale. The staining results for HIF-1α protein were classified as follows: 0, no staining; 1, nuclear staining in <1% of cells; 2, nuclear staining in 1–10% of cells and/or weak cytoplasmic staining; 3, nuclear staining in >10% of cells and/or distinct or strong cytoplasmic staining [17]. For CXCR4 protein, the membrane staining and/or cytoplasmic staining were evaluated similarly. Samples graded as 0 and 1 were considered negative, and those graded as 2 and 3 were considered positive. We used a sample of breast cancer tissue known to have strong expression of HIF-1α that had been used in a previous study [18] as a positive control and replaced primary antibodies with PBS for a negative control. The assessments were performed independently by two experienced investigators without prior knowledge of the clinical and pathological data.

### Patients and specimens

All paraffin-embedded specimens were obtained from 98 unselected cases diagnosed with OS and underwent systemic neoadjuvant chemotherapy at the Department of Orthopedic Surgery, Tangdu Hospital of the Fourth Military Medical University, between January 2003 and December 2007. The clinical stage was defined according to the 2002 American Joint Committee on Cancer (AJCC) [19].

### Statistical analysis

All statistical analyses were performed using the Statistical Package for Social Sciences software (SPSS, Inc., USA). Spearman's rank correlation was used to determine the correlation between CXCR4 and HIF-1α. The clinicopathological parameters were evaluated using univariate analysis. All p-values<0.05 were considered statistically significant.

## Results

### Hypoxia enhances the migration of SOSP-9607 cells in vitro

**Figure 1:**
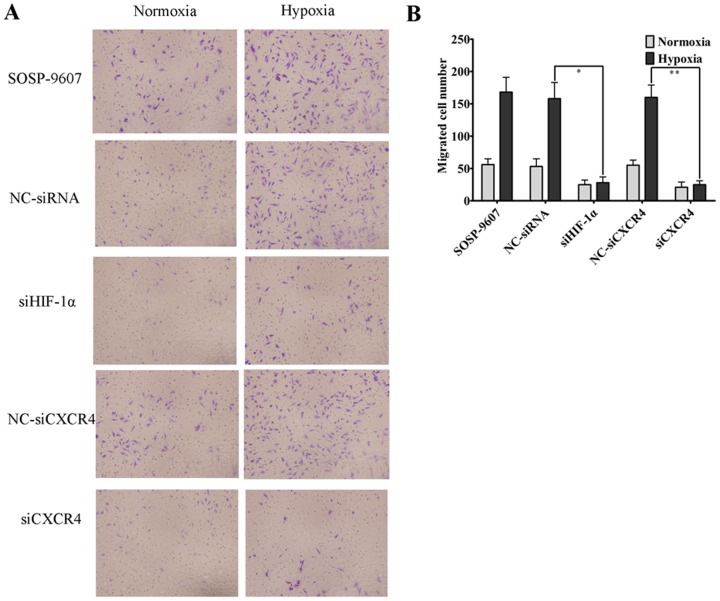
Hypoxia enhances SOSP-9607 cell migration in vitro. Human SOSP-9607 OS cells were exposed to either hypoxia or normoxia. Cell migration was assessed as described in the Methods. **A.** Representative photographs of migrated SOSP-9607 cells on the membrane at a magnification of 100×. **B.** Data are presented as the means ± SDs of duplicate samples and are representative of three independent experiments. * p<0.05, ** p<0.05.

To examine the effect of hypoxia on osteosarcoma cell metastatic ability, SOSP-9607 cells were transfected with siHIF-1α, NC-siRNA, siCXCR4 and NC-siCXCR4, and treated with normoxia or hypoxia for 48 h, then added into the top chamber of a polycarbonate membrane insert. The exposure of SOSP-9607 cells to hypoxic conditions significantly increased migration compared with normoxic conditions, whereas transfection of SOSP-9607 cells with siHIF-1α or siCXCR4 reduced the migration in hypoxic conditions compared with cells transfected with NC-siRNA or NC-siCXCR4 (Figure 1, A and B, * P<0.05, ** P<0.05).

### Hypoxia induces the expression of HIF-1α and CXCR4 in SOSP-9607 cells

**Figure 2:**
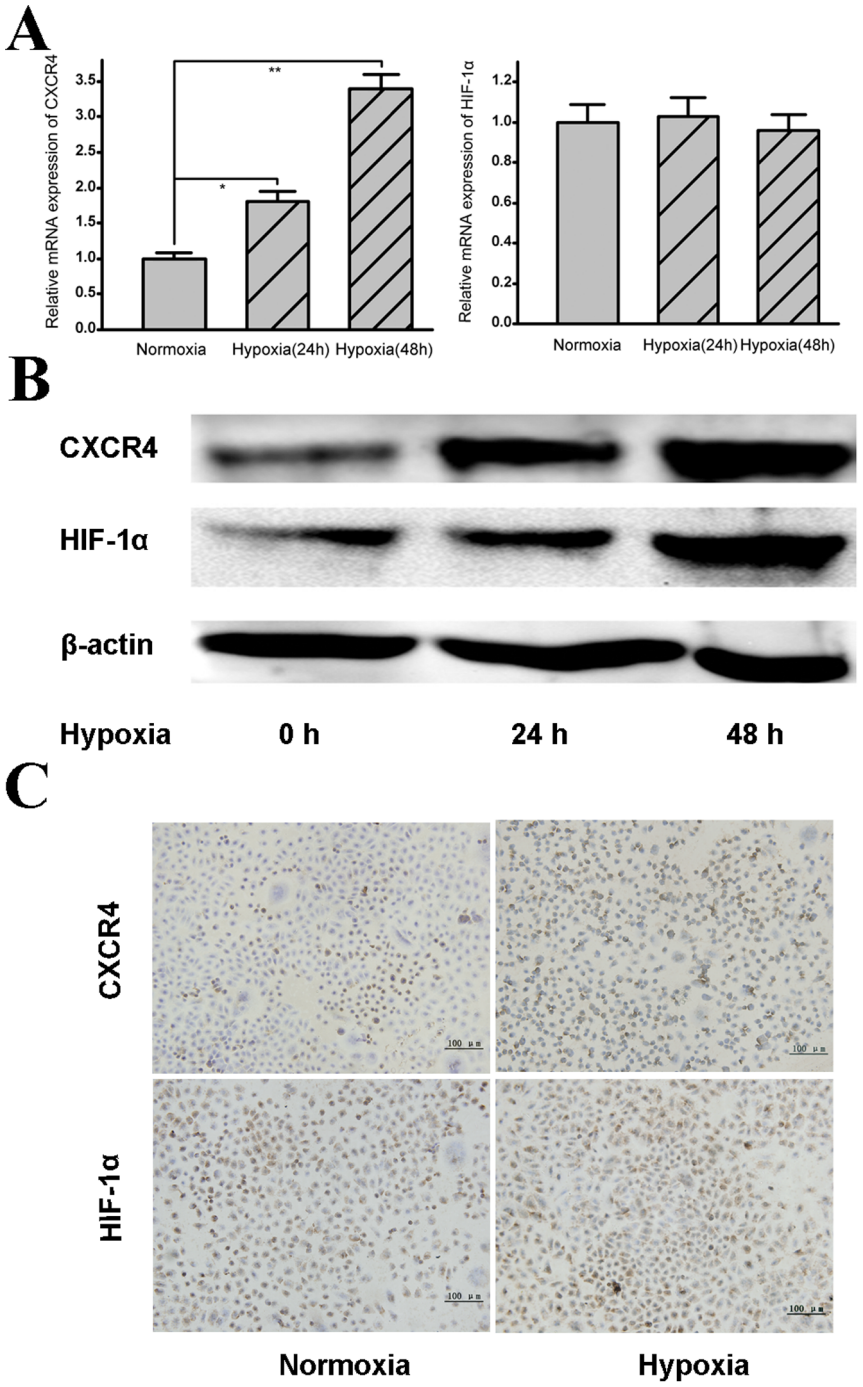
Hypoxia induces expression of HIF-1α and CXCR4 in SOSP-9607 cells. Human SOSP-9607 osteosarcoma cells were exposed to either hypoxia or normoxia. **A.** At the time points indicated, total RNA was analyzed by quantitative real-time PCR for mRNA expression of CXCR4 (left) and HIF-1α (right). Data are presented as the means ± SDs. * p<0.01, ** p<0.001. **B.** At the time points indicated, the protein levels of CXCR4 (top) and HIF-1α (middle) were analyzed by Western blotting. β-actin (bottom) was used as a control. **C.** Cells expressing CXCR4 (top) and HIF-1α (bottom) under normoxic (left) or hypoxic (right) conditions were analyzed using immunocytochemistry (200×).

Following confirmation of increased metastatic ability in SOSP-9607 cells exposed to hypoxia, we investigated whether CXCR4 plays a role in this increased migratory capacity. CXCR4 has been implicated in cancer metastases, and it has been confirmed that the expression level of CXCR4 was significantly different in various human osteosarcoma cell lines with different metastatic potentials [16]. We again treated cells with the same hypoxic and normoxic conditions and measured both the mRNA and protein levels of CXCR4 by qRT-PCR and Western blot, respectively. The results showed that the mRNA levels of CXCR4 were markedly increased under hypoxia (Figure 2A, left). The protein levels of CXCR4 were also markedly increased under hypoxic conditions in a time-dependent manner (Figure 2B, top). Moreover, we also evaluated expression of HIF-1α (a surrogate of hypoxia) at both the mRNA and protein levels. Consistent with other studies [20], no significant difference was observed in HIF-1α mRNA levels between the cells exposed to normoxia and the cells exposed to hypoxia for 24 h (Figure 2A, right, P>0.05). However, the level of HIF-1α protein was markedly increased under hypoxia in a time-dependent manner (Figure 2B, middle). Furthermore, immunocytochemistry analysis showed that the number of cells that expressed HIF-1α and CXCR4 was increased under hypoxia, and the results were statistically significant (Figure 2C).

### CXCR4 expression is regulated by HIF-1α

**Figure 3:**
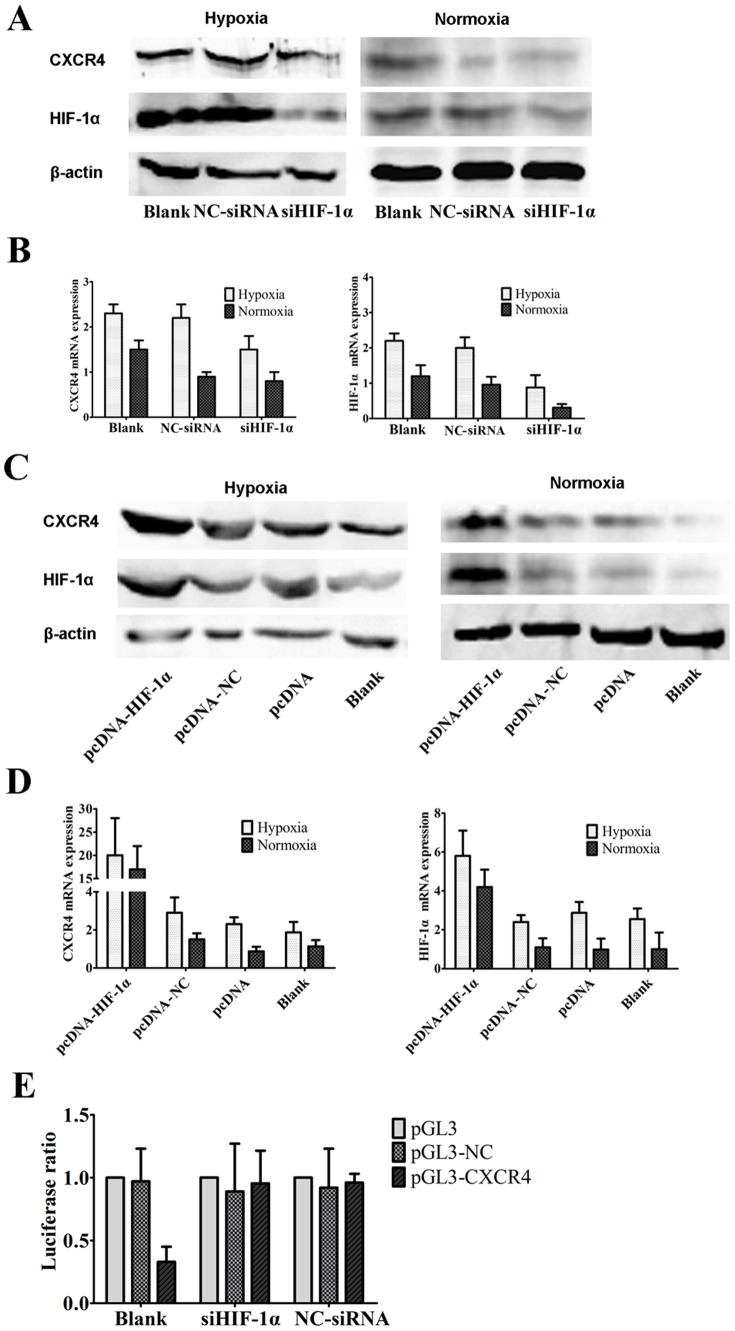
CXCR4 expression was regulated by HIF-1α. SOSP-9607 cells were transfected with siHIF-1α or NC-siRNA- and then exposed to both hypoxia and normoxia. Forty-eight hours after transfection, the protein levels of CXCR4 and HIF-1α (**A**) were analyzed by Western blot. β-actin was used as a loading control. In addition, the expression of CXCR4 and HIF-1α (**B**) mRNA was analyzed by quantitative real-time PCR. SOSP-9607 cells were transfected with pcDNA-HIF-1α or mock (pcDNA-NC) and then exposed to both hypoxia and normoxia. The pcDNA plasmid and untransfected cells (Blank) were used as a control. Forty-eight hours after transfection, the protein levels of CXCR4 and HIF-1α (**C**) were analyzed by Western blot. β-actin was used as a loading control. The expression of CXCR4 and HIF-1α (**D**) mRNA was analyzed by quantitative real-time PCR. **E.** A luciferase reporter plasmid with the CXCR4 promoter (pGL3-CXCR4) or a control (pGL3-NC) was co-transfected with siHIF-1α into SOSP-9607 cells. Twenty-four hours later, luciferase activity was measured using a dual luciferase reporter assay (Promega, USA). The results are presented as the means ± SDs of three independent experiments.

To investigate whether HIF-1α is involved in the increase of CXCR4 expression, synthetic siHIF-1α and negative control siRNA (NC-siRNA) were transfected into SOSP-9607 cells. After 48 h in normoxia and hypoxic conditions, CXCR4 and HIF-1α protein levels were markedly reduced by siHIF-1α transfection compared with NC-siRNA (Figure 3A). Moreover, the expression of CXCR4 and HIF-1α mRNA was also suppressed by siHIF-1α in SOSP-9607 cells (Figure 3CP<0.05). Additionally, we used pcDNA-HIF-1α to study the effect of HIF-1α activation on CXCR4 expression in SOSP-9607 cells. pcDNA-HIF-1α significantly increased both protein and mRNA levels of HIF-1α and CXCR4 in SOSP-9607 cells (Figure 3B and DP<0.05). Moreover, to determine whether HIF-lα affects the transcription of CXCR4 in SOSP-9607 cells, we co-transfected a luciferase reporter plasmid with the CXCR4 promoter (pGL3-CXCR4) or a control (pGL3-NC) in combination with siHIF-1α into SOSP-9607 cells. The results showed that the luciferase activity of pGL3-CXCR4 but not pGL3-NC was reduced by siHIF-1α (Figure 3E, P<0.05). These results indicated that hypoxia-induced CXCR4 expression was regulated by HIF-1α in human SOSP-9607 cells.

### Expression of HIF-1α and CXCR4 in human osteosarcoma specimens

**Figure 4:**
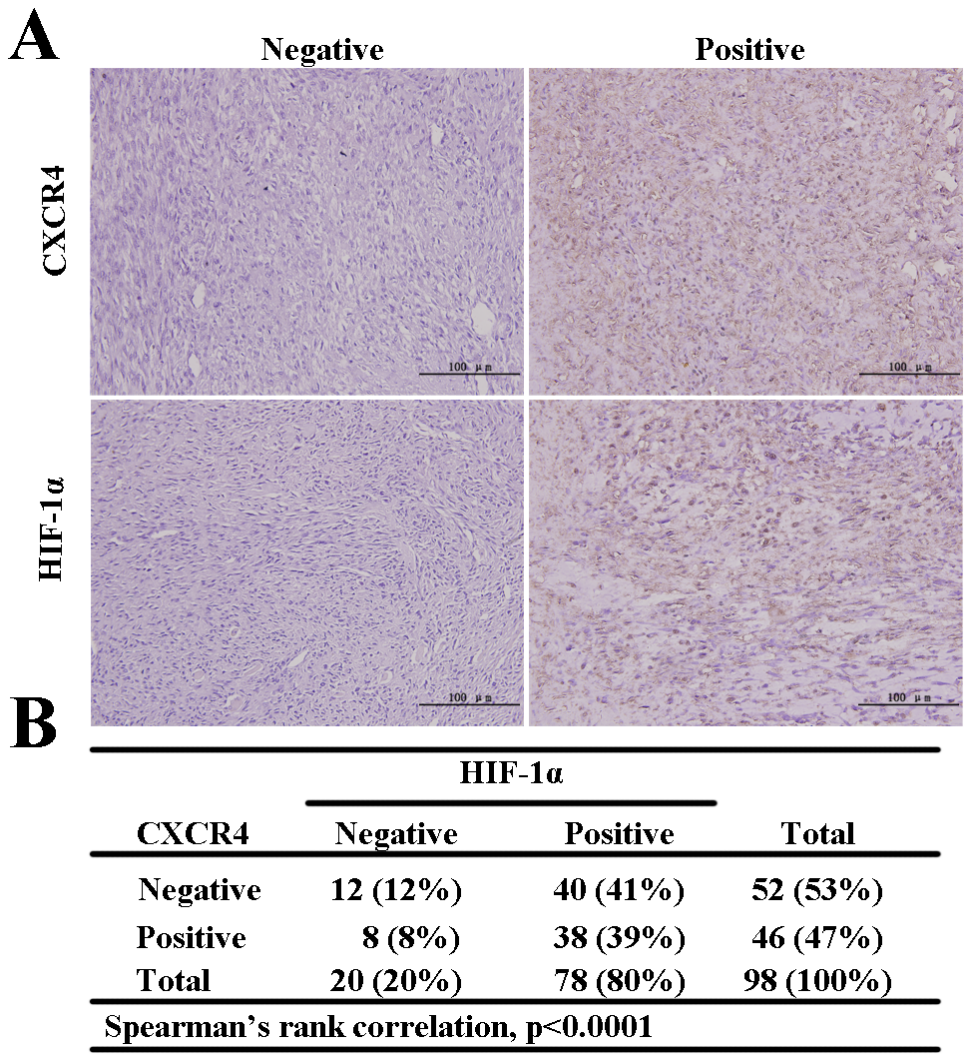
Expression of HIF-1α and CXCR4 in human osteosarcoma specimens. **A.** The levels of CXCR4 (**top**) and HIF-1α (**bottom**) were analyzed in osteosarcoma tissues with immunohistochemical staining. Representative negative (**left**) and positive (**right**) images are shown (200×). **B.** Correlation between the expression of CXCR4 and HIF-1α in human osteosarcoma. Spearman's rank correlation, p<0.001.

Having demonstrated that hypoxia-induced CXCR4 expression was regulated by HIF-1α in osteosarcoma cell lines, we next investigated the correlation between CXCR4 and HIF-1α expression in 98 osteosarcoma cases. Immunohistochemistry results showed that 78 cases expressed HIF-1α mainly in the nucleus but also in the cytoplasm. Forty-six cases expressed CXCR4 on the cell membrane and in the cytoplasm. Representative images of CXCR4 and HIF-1α are shown in Figure 4A. Thirty-eight of the 46 samples (83%) that expressed CXCR4 also expressed HIF-1α (Figure 4B), and this association was highly significant (p<0.001, Spearman's rank correlation). Univariate analysis revealed that expression of these proteins was correlated with clinical stage, metastasis and survival but not age, gender or tumor size. The patients with tumors expressing CXCR4 and HIF-1α had lower overall survival rates compared with patients with tumors that did not express CXCR4 or HIF-1α (Table 1). These data indicated that CXCR4 and HIF-1α expression is highly correlated with metastatic progression in patients with OS and had predictive value for the metastasis and survival of OS patients.

**Table 1:** Distribution of clinicopathological parameters for the 98 primary osteosarcoma patients.

Parameter	Category	Frequency (n)	Percent (%)	CXCR4 (positive)	P	HIF-1α (positive)	P
Gender	Female	39	40	19	0.774	32	0.623
	Male	59	60	27		46	
Age	≤20	74	74	34	0.729	65	3.1428×10^−10^
	>20	24	24	12		13	
Anatomic location	Femur/tibia	67	68	38	0.004	65	3.1429×10^−10^
	Elsewhere	31	32	8		13	
Histological Type	Osteoblastic (O)	70	71	29	0.252	55	0.772
	Chondroblastic (C)	9	9	5		8	
	Fibroblastic	9	9	4		6	
	Osteoclast rich	6	6	4		5	
	Telangiectasia	2	2	2		2	
	Not classifiable	2	2	2		2	
Tumor Size* (cm^3^)	≤50	43	44	24	0.12	41	0.001
	>50	55	56	22		38	
Clinical stage	IIA/IIB	59	60	8	3.76×10^−15^	39	0.000247
	III	20	20	20		20	
	IVA	19	20	18		19	
Metastases (lung)	Yes	41	42	38	1.41×10^−14^	40	0.000182
	No	57	58	8		38	
Survival	Died	19	19	15	0.002	19	0.014
	Alive	79	81	31		59	

## Discussion

Our findings confirm that many OS specimens express HIF-1α and suggest that hypoxia is a common character of OS. Meanwhile, the level of HIF-1α expression in SOSP-9607 cells significantly increased under hypoxic conditions. Hypoxia is a common phenomenon in human solid cancers and is associated with tumor development, invasion and metastasis. Tumor progression requires an increased adaptation to a hypoxic microenvironment [21]. As a major regulator of the cellular response to hypoxia, the HIF-1α subunit is detectable in the nucleus of normoxic cells yet undetectable in most cell types due to its rapid degradation by the ubiquitin-proteasome system [22]. Overexpression of HIF-1α is also a good indicator for poor response to chemoradiotherapy [23]. Inhibition of HIF-1α expression may lead to inhibition of cell proliferation and growth, induction of apoptosis, and prevention of tumor progression [24]–[26]. Previous studies have indicated that activation of HIF-1α occurs in many solid tumors. In the present study, we found that higher HIF-1α expression was associated with poor prognosis, which is consistent with the results of Yang QC et al. [27].

Tumor progression, especially tumor metastasis, is also affected by CXCR4-SDF-1 signaling through the induction of tumor-associated integrin activation and signaling [28]. The role of CXCR4 and its unique ligand SDF-1α has been investigated in organ-specific metastases of several types of cancer, including breast cancer, and hypoxia increased the metastatic ability of breast cancer cells via upregulation of CXCR4 [14]. Huang CY found that human OS cell lines had significantly increased expression of SDF-1 and CXCR4 [29]. Our previous study confirmed that the expression level of CXCR4 was significantly different in various OS cell lines with pulmonary metastatic potential [30]. Therefore, we analyzed the correlation between the high expression of both HIF-1α and CXCR4 and clinicopathologic significance in human OS samples. We found that coexpression of HIF-1α and CXCR4 was significantly positively correlated with poor prognosis in human OS specimens. However, we observed that 40% of HIF-1α-positive samples were CXCR4-negative. Previous data suggested that overexpression of HIF-1α might occur very early in carcinogenesis, before histological evidence of angiogenesis or invasion [31]. Furthermore, it was postulated that certain hypoxia-regulated RNA binding factors might interact and stabilize CXCR4 mRNA at the posttranscriptional level [32]. Therefore, we considered whether the more than 40% of cases without CXCR4 expression might result from the lack of additional mechanisms of control needed for CXCR4 stability in cancer tissue. Daniel J Ceradini demonstrated that HIF-1-induced SDF-1 expression increased the adhesion, migration and homing of circulating CXCR4-positive progenitor cells to ischemic tissue. Discrete regions of hypoxia in the bone marrow compartment also increased SDF-1 expression and progenitor cell tropism [33]. These data showed that the recruitment of CXCR4-positive progenitor cells to regenerating tissues was mediated by hypoxic gradients via HIF-1-induced expression of SDF-1. Hypoxia contributes to the progression of a variety of cancers by activating adaptive transcriptional programs that promote cell survival, motility and tumor angiogenesis. Organ-specific metastasis is governed, in part, by interactions between chemokine receptors on cancer cells and the matching chemokines in target organs. Therefore, a previous study showed the expression of a receptor important for homing to distant organs was directly regulated by the hypoxia pathway and that its upregulation was already observed in early tumor stages. The propensity to metastasize, at least for certain cancers, might therefore be determined by the identities of the mutant alleles acquired relatively early during multistep tumorigenesis [34]. Interestingly, Knowles HJ demonstrated that OS cells showed a trend toward increased hypoxic migration and responded to HIF-siRNA, with targeted inhibition of HIF-2α resulting in increased cell migration under hypoxia [35]. However, in our study, hypoxia upregulated the transcription of the CXCR4 gene by enhancing the transcription factor HIF-1α and thus promoted cell migration in human osteosarcoma. In contrast, si-HIF-1α decreased SOSP-9607 cell migration. This result suggests that cell migration may be dependent on either HIF-1α and/or HIF-2α in different cell types and HIF-1α and HIF-2α may exert distinct phenotypic effects in different cell lines [36].

In conclusion, this research reports the effect of hypoxia on HIF-1α expression and cell migration and the correlation of HIF-1α with CXCR4 in osteosarcoma cells. Although we are currently unable to dissect the specific mechanisms by which HIF-1α upregulates CXCR4 in vivo, we have identified that the hypoxia-HIF-1α-CXCR4 pathway may regulate trafficking and localization of SOSP-9607 cells and may represent a target for novel therapeutic strategies for human osteosarcoma.
